# Multimodal MRI radiomics-based stacking ensemble learning model with automatic segmentation for prognostic prediction of HIFU ablation of uterine fibroids: a multicenter study

**DOI:** 10.3389/fphys.2024.1507986

**Published:** 2024-12-20

**Authors:** Bing Wen, Chengwei Li, Qiuyi Cai, Dan Shen, Xinyi Bu, Fuqiang Zhou

**Affiliations:** ^1^ Department of Radiology, Yiyang Central Hospital, Yiyang, China; ^2^ Department of Radiology, The Third People’s Hospital of Chengdu, Chengdu, China

**Keywords:** artificial intelligence, uterine fibroids, high intensity focused ultrasound, ensemble stacking model, magnetic resonance imaging

## Abstract

**Objectives:**

To evaluate the effectiveness of an MRI radiomics stacking ensemble learning model, which combines T2-weighted imaging (T2WI) and contrast-enhanced T1-weighted imaging (CE-T1WI) with deep learning-based automatic segmentation, for preoperative prediction of the prognosis of high-intensity focused ultrasound (HIFU) ablation of uterine fibroids.

**Methods:**

This retrospective study collected data from 360 patients with uterine fibroids who underwent HIFU treatment. The dataset was sourced from Center A (training set: N = 240; internal test set: N = 60) and Center B (external test set: N = 60). Patients were categorized into favorable and unfavorable prognosis groups based on the post-treatment non-perfused volume ratio. Automated segmentation of uterine fibroids was performed using a V-net deep learning models. Radiomics features were extracted from T2WI and CE-T1WI, followed by data preprocessing including normalization and scaling. Feature selection was performed using *t*-test, Pearson correlation, and LASSO to identify the most predictive features for preoperative prognosis Support Vector Machine (SVM), Random Forest (RF), Light Gradient Boosting Machine (LightGBM), and Multilayer Perceptron (MLP) were employed as base learners to construct base predictive models. These models were integrated into a stacking ensemble model, with Logistic Regression serving as the meta-learner to combine the outputs of the base models. The performance of the models was assessed using the area under the receiver operating characteristic curve (AUC).

**Results:**

Among the base models developed using T2WI and CE-T1WI features, the MLP model exhibited superior performance, achieving an AUC of 0.858 (95% CI: 0.756–0.959) in the internal test set and 0.828 (95% CI: 0.726–0.930) in the external test set. It was followed by the SVM, LightGBM, and RF, which obtained AUC values of 0.841 (95% CI: 0.737–0.946), 0.823 (95% CI: 0.711–0.934), and 0.750 (95% CI: 0.619–0.881), respectively. The stacking ensemble learning model, which integrated these five algorithms, demonstrated a notable enhancement in performance, with an AUC of 0.897 (95% CI: 0.818–0.977) in the internal test set and 0.854 (95% CI: 0.759–0.948) in the external test set.

**Conclusion:**

The DL based automatic segmentation MRI radiomics stacking ensemble learning model demonstrated high accuracy in predicting the prognosis of HIFU ablation of uterine fibroids.

## 1 Introduction

Uterine fibroids are the most prevalent benign tumors of the female reproductive system, characterized by high vascularity, with incidence rates increasing with age. The symptoms associated with fibroids can appreciably impair women’s quality of life (Stewart et al., 2017; Bulun, 2013). Traditional treatment modalities primarily encompass hysterectomy, myomectomy, laparoscopic myomectomy, and uterine artery embolization (Lethaby et al., 2017; Donnez et al., 2015; Jacoby et al., 2020; McLucas et al., 2001). Recently, non-invasive high-intensity focused ultrasound (HIFU) has gained prominence in the management of symptomatic uterine fibroids and is now included in treatment guidelines in several countries, with its effectiveness extensively corroborated (Lyon et al., 2020; Chen et al., 2018; Liu et al., 2020). Nevertheless, due to the inherent properties of fibroid tissue and technical constraints, HIFU may not be suitable for all patients (Huang et al., 2019; Zhang et al., 2020; Verpalen et al., 2019; Verpalen et al., 2020). Therefore, precise preoperative evaluation is imperative for the successful application of HIFU. Enhancing the accuracy of preoperative predictions regarding HIFU treatment efficacy is crucial for clinicians to formulate optimal treatment strategies.

Preoperative magnetic resonance imaging (MRI) with HIFU is instrumental in the differential diagnosis of uterine fibroids, as well as in assessing their location, morphology, and potential tissue composition (Liao et al., 2023; Venkatesan et al., 2012). MRI is recommended as an essential preoperative evaluation tool for HIFU treatment of uterine fibroids. The imaging characteristics of fibroids across various MRI sequences exhibit a notable correlation with their tissue heterogeneity. Pervious research has elucidated that T2-weighted imaging (T2WI) offers superior visualization of the internal architecture and morphology of uterine fibroids, facilitating the differentiation of cellular hydration levels from fibrous tissue composition (Zhao et al., 2013; Zhao et al., 2015; Funaki et al., 2007). Moreover, contrast-enhanced T1-weighted imaging (CE-T1WI), achieved through the administration of contrast agents, augments the delineation of fibroid boundaries relative to the surrounding tissues, thereby enhancing the assessment of vascularization (Keserci and Duc, 2017; Yoon et al., 2010). Consequently, it assists in evaluating the proliferative activity and invasive potential of the fibroids. These imaging characteristics are instrumental in enabling clinicians to subjectively assess the tissue characteristics of fibroids and forecast the efficacy of ultrasound energy in inducing coagulative necrosis in the targeted region. Despite these advances, challenges remain due to the variability in clinical experience and the inherent limitations of human visual assessment in accurately evaluating fibroid morphology, spatial distribution, and lesion characteristics (Dou et al., 2024).

Radiomics is revolutionizing medical imaging by enabling the detailed analysis of tumor complexity at a microscopic level through technological advancements (Guiot et al., 2022; Gillies et al., 2016). By utilizing high-throughput imaging data, radiomics overcomes the limitations of traditional imaging, revealing subtle features that are often imperceptible to the naked eye (Lambin et al., 2017). This cutting-edge technology not only enhances the precision of tumor diagnosis but also facilitates personalized treatment, providing unparalleled insight into the potential threats posed by tumors. In the preoperative assessment of HIFU ablation of uterine fibroids, radiomics models have demonstrated significant clinical potential. However, previous studies have primarily relied on radiomics features from single MRI sequences for efficacy prediction (Cheng et al., 2024; Li et al., 2023). While some progress has been achieved, single sequences often fail to comprehensively represent the biological information of fibroids, leading to limited predictive accuracy. By integrating information from different MRI sequences, a more comprehensive understanding of the tissue characteristics and biological properties of fibroids can be achived, which significantly enhancing the accuracy and stability of prediction models (Zheng et al., 2021). Nonetheless, existing research has not fully incorporated the CE-T1WI sequence, which most accurately reflects fibroid blood supply, and has overlooked the importance of including diverse MRI sequences in the model to enhance its performance. To facilitate this integration, deep learning (DL) based automatic segmentation plays a crucial role by providing precise delineation of fibroid boundaries and relevant anatomical structures across multiple MRI sequences (Wang et al., 2014). As Imran Iqbal demonstrated, deep learning can effectively extract disease-related information and address challenges such as limited annotated data by leveraging pre-trained models, which has proven beneficial in detecting various medical conditions, including joint abnormalities and skin cancer screening (Lqbal et al., 2021; Iqbal et al., 2020). Such methodologies not only enhance the accuracy of segmentation but also provide a solid foundation for the subsequent extraction of radiomic features, ensuring both the efficiency and consistency of the analysis. The application of automated segmentation techniques allows researchers to rapidly process large volumes of imaging data, thereby improving the reliability and predictive power of model construction. However, in the realm of machine learning applications, traditional single algorithms and hyperparameter tuning methods, although effective, may not fully exploit the potential of the radiomics features. Stacking, as an advanced ensemble learning method, demonstrates substantial potential in enhancing predictive performance (Naimi and Balzer, 2018). Stacking first trains multiple types of base learners to capture diverse features and patterns within the data. Subsequently, the outputs from these base learners are used as inputs to train a meta-learner, which integrates the strengths of the base learners and addresses their shortcomings, ultimately producing more accurate results. This method leverages the advantages of multiple models to effectively handle complex data, reduce overfitting, and improve prediction accuracy and stability (Sesmero et al., 2015). This study aims to develop a multimodal MRI radiomics analysis method that integrates automated segmentation and stacking ensemble learning techniques to further enhance the predictive performance of HIFU ablation of uterine fibroids.

## 2 Materials and methods

### 2.1 Patients

**FIGURE 1:**
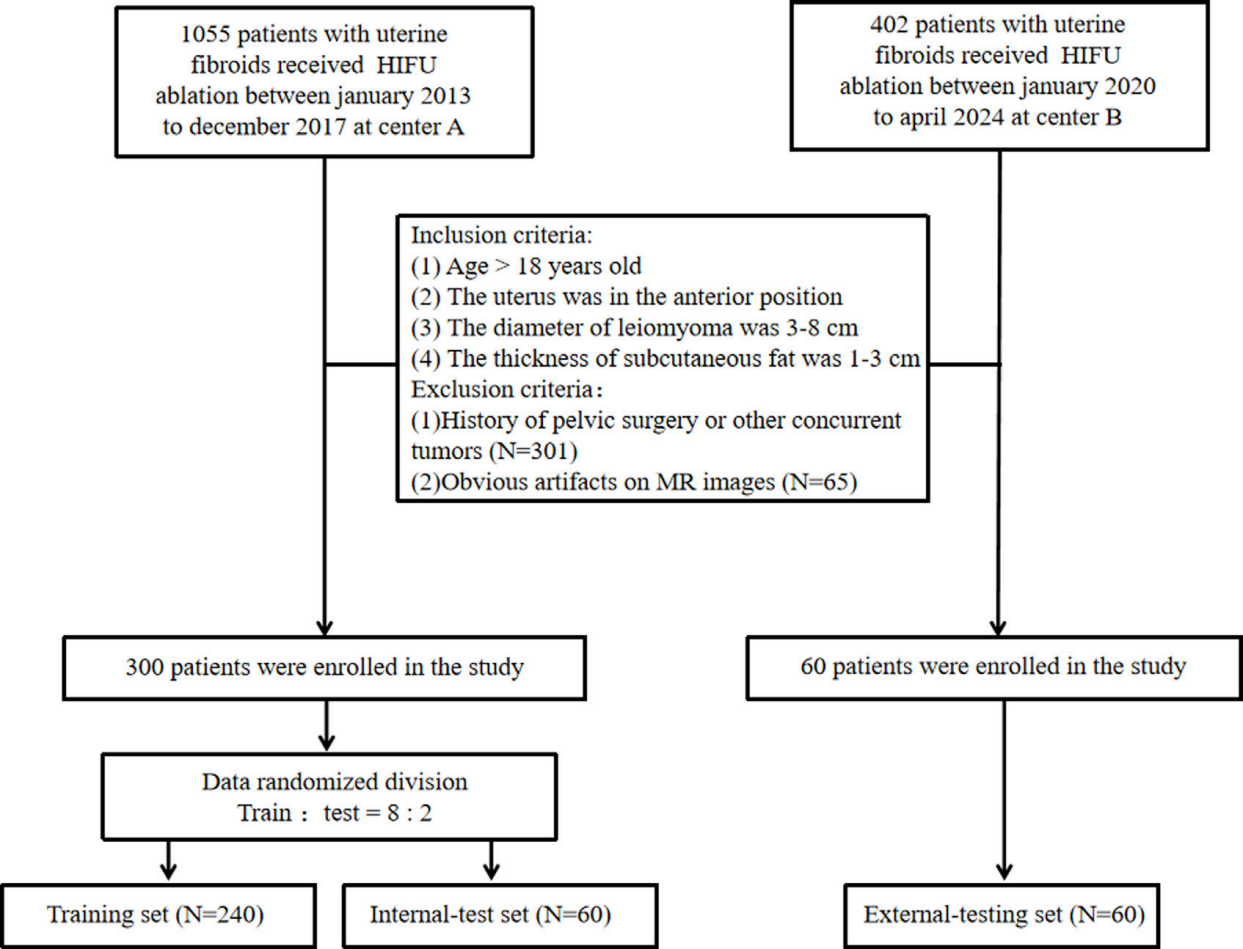
Flowchart of patient enrollment and exclusion.

We conducted a retrospective analysis involving 1457 consecutive patients diagnosed with uterine fibroids across two medical centers. To accurately assess the efficacy of HIFU ablation for uterine fibroids while controlling for various factors on efficacy, such as fibroid size, location, tissue composition, abdominal wall thickness, and the presence of abdominal wall scars. We established the following inclusion criteria (Stewart et al., 2017): patients over 18 years old (Bulun, 2013); no prior surgical interventions or relevant medication history (Lethaby et al., 2017); fibroids located in the anterior uterine position (Donnez et al., 2015); fibroids measuring between 3–8 cm in diameter (Jacoby et al., 2020); abdominal fat thickness ranging from 1 to 3 cm; and (McLucas et al., 2001) for patients with multiple fibroids, only the largest fibroid was included. The exclusion criteria were (Stewart et al., 2017): history of pelvic surgery or other concurrent tumors; and (Bulun, 2013) imaging artifacts that interfered with accurate assessment. A non-perfused volume ratio (NPVR) ≥80% was used to define treatment efficacy, a threshold validated across different levels of physician expertise (Gong et al., 2022). Thus, an NPVR of ≥80% was considered indicative of a favorable prognosis, while an NPVR <80% was deemed an unfavorable prognosis. NPVR assessments were independently performed by two radiologists: one with 4 years and the other with 15 years of diagnostic experience. Discrepancies were resolved in favor of the more experienced radiologist. After a stringent screening process, we included 300 patients from Center A and 60 from Center B, dividing them into three groups: a training set (N = 240), an internal test set (N = 60), and an external test set (N = 60). Approval for the study was granted by the Institutional Review Boards of both centers (Center A: approval number 2022-K129, Center B: approval number 2024-J-29), and the need for written informed consent was waived. The patient enrollment process is detailed in Figure 1.

### 2.2 Images acquisition

MRI scans were acquired from two centers: one using a 3.0 T Signa HDxt MRI scanner and the other using a 1.5 T Signa Voyager MRI scanner, both provided by General Electric. Patients were positioned in the supine position and scanned using a dedicated 8-channel phased array coil for the abdomen. Detailed MRI acquisition parameters are shown in Table 1.

**TABLE 1:** MRI acquisition parameters.

Parameters	Signa HDxt		Signa voyager	
	T2WI	CE-T1WI	T2WI	CE-T1WI
Magnetic field strength	3.0 T		1.5 T	
Repetition time (TR)	270	3.84	4,000	6
Echo time (TE)	2.1	1.81	68	2
Feld of view (FOV)	98.1 × 38	68.4 × 26.5	60 × 60	60 × 60
Slice thickness (mm)/gap (mm)	6/2	4/2	6/2	4/2
matrix	512 × 512	512 × 512	256 × 256	256 × 256

### 2.3 Image segmentation and feature extraction

**FIGURE 2:**
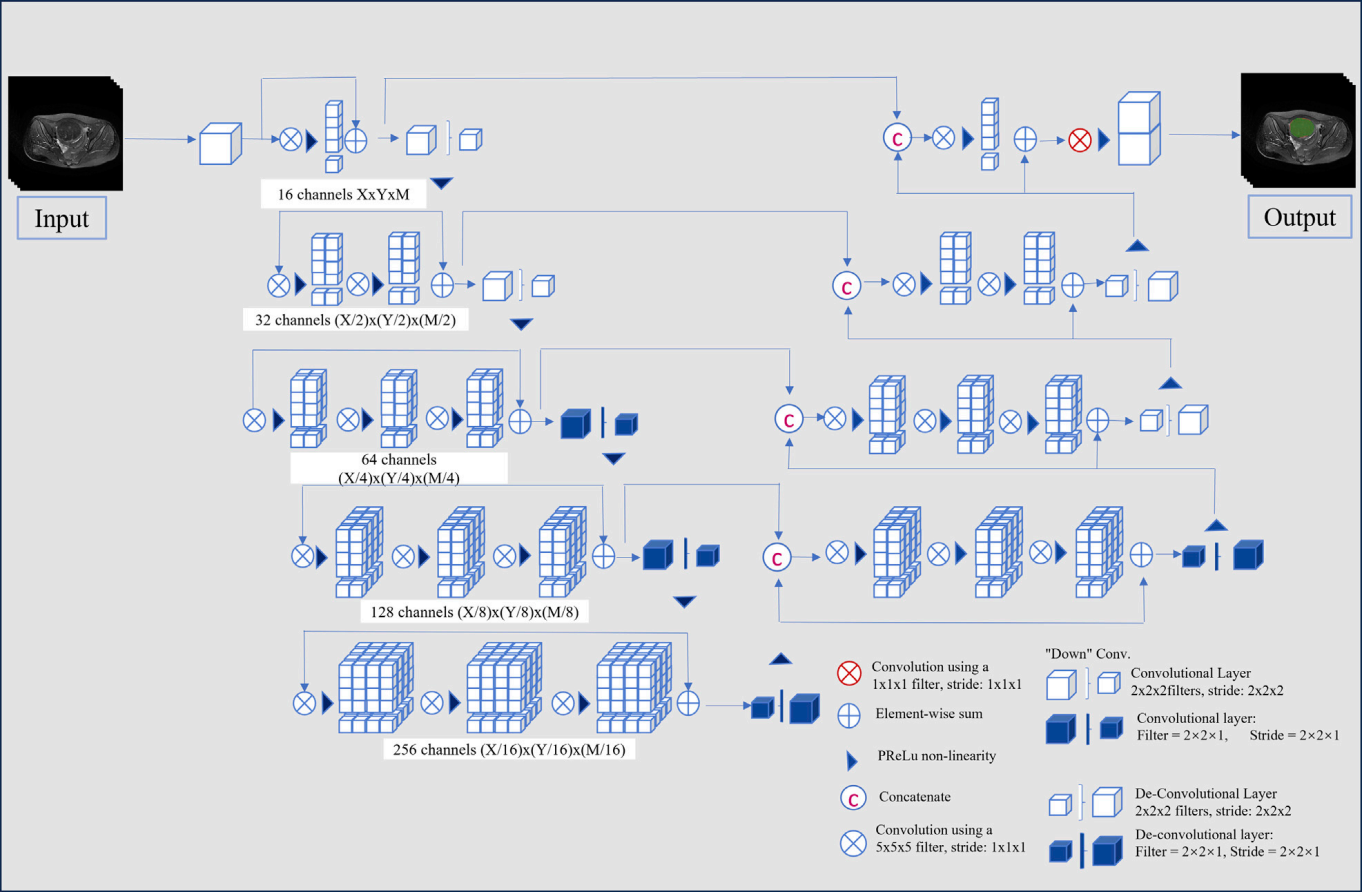
Schematic diagram of the segmentation model structure.

**FIGURE 3:**
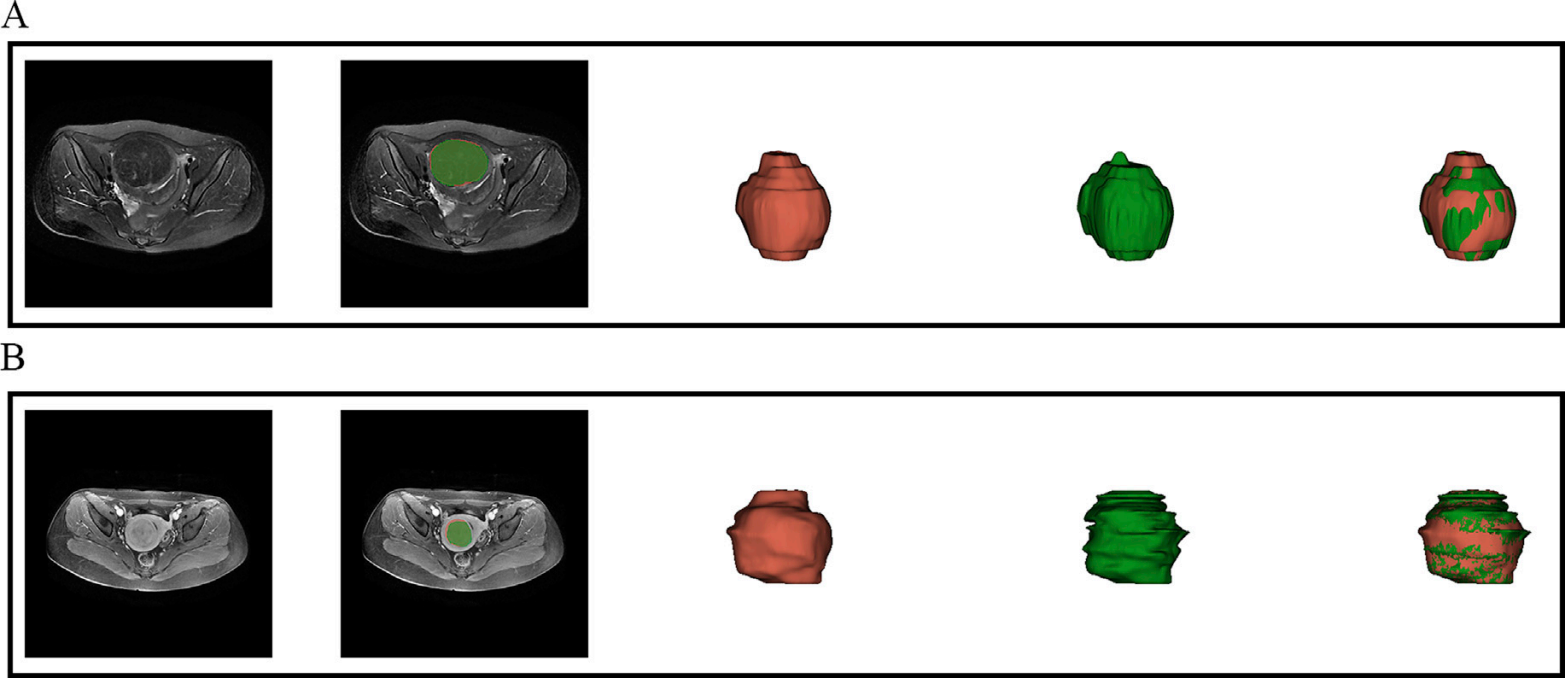
Visual inspection of the segmentation results. **(A)** presents the automatic segmentation results on T2WI for a 48-year-old patient diagnosed with uterine fibroids, while **(B)** presents the automatic segmentation results on CE-T1WI for a 39-year-old patient also diagnosed with uterine fibroids.

This study employed a V-Net architecture for automatic segmentation of pelvic MRI data, specifically targeting uterine fibroids in T2WI and CE-T1WI (Milletari et al., 2016). The network is based on an encoder-decoder architecture. The encoder uses 3D convolutional modules to extract features from medical images and adjusts the feature resolution through convolution operations with a stride of 2. In the decoder, 3D transposed convolutions are used to progressively restore the deep semantic features extracted by the encoder to a higher resolution. Skip connections are incorporated between the encoder and decoder to effectively combine low-level and high-level features, thereby enhancing segmentation accuracy. The network includes three auxiliary loss layers and one main loss layer; the main loss layer employs 3D transposed convolutions to recover the feature maps to the original image size, ultimately outputting the automatically segmented target regions. Figure 2 illustrates the architecture of the automatic segmentation network, while Figure 3 presents the segmentation results. We evaluated the model’s performance on the validation data set, which yielded an average Dice Similarity Coefficient (DSC) of 0.883 for the T2WI segmentation model and 0.809 for the CE-T1WI segmentation model, indicating good performance in the automatic segmentation for uterine fibroids.

After the automatic segmentation of the remaining MRI scans, two independent radiologists evaluated the segmented regions. For images with inaccurate outlines, manual corrections were made using ITK-SNAP software (version 3.8, http://www.itksnap.org) to create complete Regions of Interest (ROIs). Prior to feature extraction, all MRI images and segmentations were resampled to a voxel size of 1 × 1 × 1 mm^3^ using bilinear interpolation. Features were extracted from the T2WI and CE-T1WI ROIs using Python (version 3.10, https://www.python.org), focusing on both low-dimensional and high-dimensional aspects. Low-dimensional features comprised shape and first-order histogram metrics, while high-dimensional features included texture characteristics derived from various matrices: gray-level co-occurrence matrix (GLCM), gray-level run-length matrix (GLRLM), gray-level size-zone matrix (GLSZM), neighborhood gray-tone difference matrix (NGTDM), and gray-level dependence matrix (GLDM). Additionally, texture features were analyzed in the Gaussian Laplacian filter domain (core sizes ranging from 2.0 to 5.0 mm) and the wavelet filter domain.

### 2.4 Feature selection

To assess the interobserver repeatability of radiomic features, the intraclass correlation coefficient (ICC) was calculated using data from 100 randomly selected patients at center A. Features with ICC values greater than 0.8 were deemed highly consistent and were included for further analysis. To harmonize radiomic features across different MRI scanners, the ComBat method was applied (Orlhac et al., 2022). To address biases introduced by imbalanced data distributions, which could affect model performance, the Synthetic Minority Over-sampling Technique (SMOTE) was applied to increase the number of samples in underrepresented classes, thereby improving model robustness (Chawla et al., 2002). Subsequently, feature selection was conducted on the harmonized T2WI and CE-T1WI features. Initially, a *t*-test was employed to filter features with significant correlations to treatment outcomes, retaining those with strong associations. Pearson’s correlation coefficient was then computed to analyze the relationships among these features, retaining features with coefficients above 0.8. Finally, the Least Absolute Shrinkage and Selection Operator (LASSO) regression was applied for further refinement, aiming to reduce dimensionality and select the most relevant features for analysis.

### 2.5 Construction of stacking ensemble learning model

**FIGURE 4:**
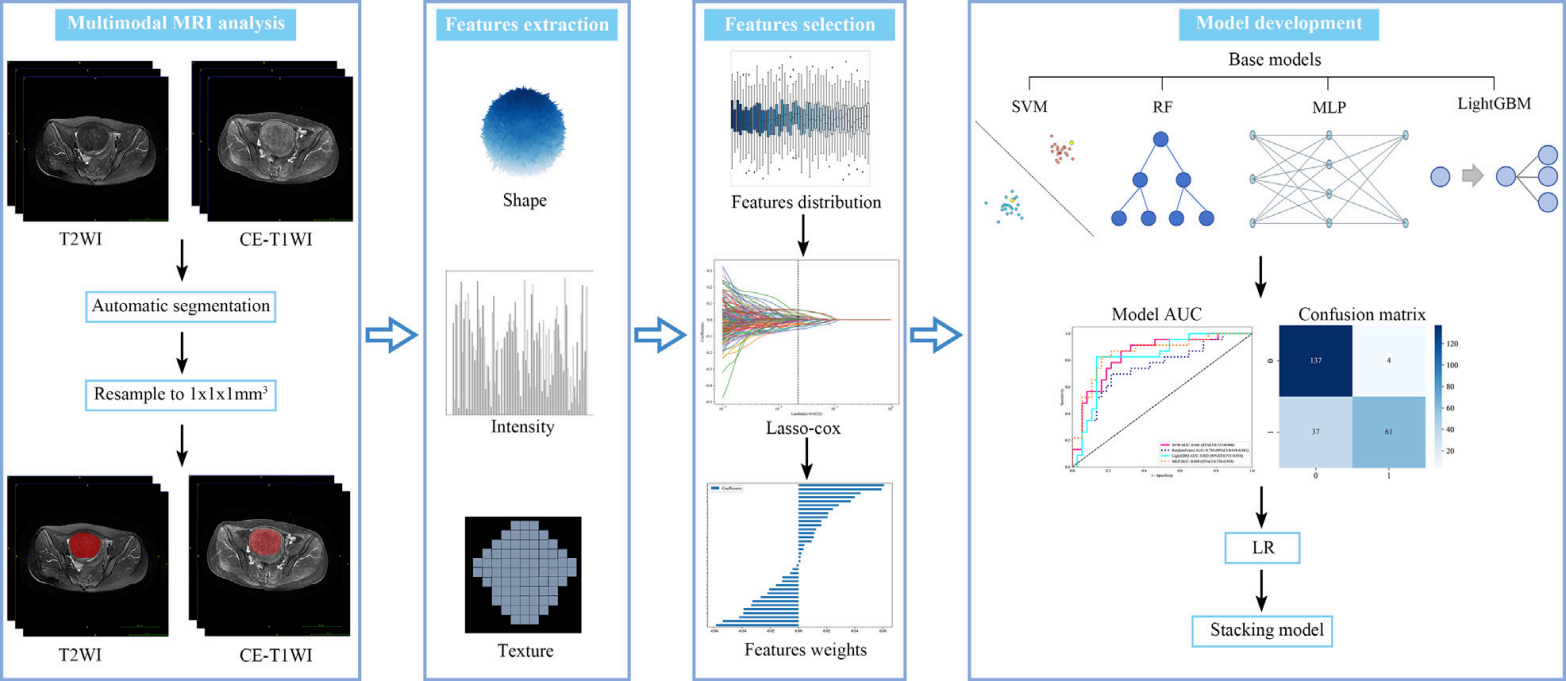
Workflow of the radiomics analysis in this study.

Stacking ensemble learning is an advanced machine learning technique that combines the outputs of multiple base learners using a meta-learner to improve predictive performance. In this study, four traditional machine learning algorithms were employed as base learners: Support Vector Machine (SVM), Random Forest (RF), Multilayer Perceptron (MLP), and Light Gradient Boosting Machine (LightGBM). These base learners formed the first layer of the ensemble model. A Logistic Regression (LR) algorithm was selected as the meta-learner for the second layer due to its strong generalization capabilities and effectiveness in combining the predictions of the base learners, while addressing their biases and reducing overfitting. Hyperparameters for each base learner were optimized using Bayesian optimization, which efficiently searches the hyperparameter space to enhance model performance (Snoek et al., 2012). The predictive performance of the ensemble model was evaluated by calculating the area under the receiver operating characteristic curve (AUC). In addition, metrics such as accuracy, sensitivity, and specificity were assessed using the maximum Youden’s index to provide a comprehensive evaluation of model performance. The radiomics analysis pipeline is shown in Figure 4.

### 2.6 Statistical analysis

All statistical analysis was performed using SPSS (version 26.0) and Python (version 3.10). The kappa test was used to analyze the consistency of patient grouping results between two radiologists (Kappa values in the range of 0.80–1.00, good consistency; 0.40 to 0.79, fair consistency; less than 0.40, poor consistency). Continuous variables were described as mean ± standard deviation and compared by a Mann–Whitney *U* test or *t*-test. Categorical variables were summarized as frequencies and percentages using the chi-square test or Fisher’s exact test. The AUC of different models were statistically compared using the DeLong test. A P-value <0.05 was considered statistically significant.

## 3 Results

### 3.1 Patient characteristics and outcome

In the study, a total of 354 patients were initially enrolled at Center A. Out of these, 54 patients were excluded due to either incomplete or artifact-ridden imaging data. The remaining cohort comprised 172 patients with a favorable prognosis and 128 patients with an unfavorable prognosis. At Center B, 65 patients were screened, with 5 exclusions for the same reasons. This left 35 patients with a favorable prognosis and 25 with an unfavorable prognosis. The inter-rater reliability between the two radiologists was evaluated using the Kappa statistic, which yielded a value of 0.894 (P < 0.001), reflecting a strong level of agreement. The clinical characteristics of the patients are detailed in Table 2.

**TABLE 2:** Clinical and radiological characteristics of patients.

Characteristics	Traning set (n = 240)		P-Value	Internal test set (N = 60)		P-Value	External test set (N = 60)		P-Value
	NPVR ≥ 80% (n = 137)	NPVR < 80% (n = 103)		NPVR ≥ 80% (n = 35)	NPVR < 80% (n = 25)		NPVR ≥ 80% (n = 38) NPVR < 80% (n = 25)	NPV < 80% (n = 22) NPVR < 80% (n = 25)	
Age (years)	39.63 ± 6.22	39.22 ± 6.17	0.496	39.69 ± 6.83	38.36 ± 7.33	0.475	38.18 ± 8.43	40.47 ± 7.67	0.287
Abdominal Fat Thickness (mm)	16.02 ± 7.63	17.11 ± 9.62	0.108	15.89 ± 5.41	17.08 ± 6.31	0.776	20.44 ± 8.71	18.84 ± 5.94	0.69
Fibroid Type (n (%))			<0.001			0.306			0.165
Submucosal	3 (2.19)	2 (1.94)		1 (2.86)	2 (8.00)		3 (7.89)	0 (0.00)	
Intramural	115 (83.94)	57 (55.34)		32 (91.43)	16 (64.00)		23 (60.53)	18 (81.82)	
Subserosal	19 (13.87)	44 (42.72)		2 (5.71)	7 (28.00)		12 (31.58)	4 (18.18)	
T2WI Signal Intensity			0.809			0.141			0.984
Hypointensity	74 (54.01)	57 (55.34)		15 (42.86)	12 (48.00)		29 (76.32)	17 (77.27)	
Isointensity	27 (19.71)	17 (16.50)		2 (5.71)	5 (20.00)		5 (13.16)	3 (13.64)	
Hyperintensity	36 (26.28)	29 (28.16)		18 (51.43)	8 (32.00)		4 (10.53)	2 (9.09)	
T2WI Signal Homogeneity			0.044			1.000			0.951
Homogeneous	98 (71.53)	60 (58.25)		20 (57.14)	15 (60.00)		16 (42.11)	10 (45.45)	
Inhomogeneous	39 (28.47)	43 (41.75)		15 (42.86)	10 (40.00)		22 (57.89)	12 (54.55)	
CE-T1WI Signal Intensity			<0.001			0.006			0.359
Hypointensity	70 (51.09)	67 (65.05)		17 (48.57)	19 (76.00)		21 (55.26)	8 (36.36)	
Isointensity	49 (35.77)	5 (4.85)		14 (40.00)	1 (4.00)		8 (21.05)	6 (27.27)	
Hyperintensity	18 (13.14)	31 (30.10)		4 (11.43)	5 (20.00)		9 (23.68)	8 (36.36)	
CE-T1WISignal Homogeneity			0.869			0.964			0.816
Homogeneous	44 (32.12)	35 (33.98)		14 (40.00)	11 (44.00)		8 (21.05)	6 (27.27)	
Inhomogeneous	93 (67.88)	68 (66.02)		21 (60.00)	14 (56.00)		30 (78.95)	16 (72.73)	

### 3.2 Feature selection

Initially, 2,394 features were initially extracted from the ROIs in T2WI and CE-T1WI. After applying an ICC threshold of 0.8, 2,376 features were retained for further analysis. To address class imbalance, the SMOTE algorithm was employed to increase the number of unfavorable prognosis cases in Center A by 44 instances. The *t*-test was performed to identify features significantly associated with HIFU prognosis, resulting in 491 features. Then, Pearson correlation analysis was used to filter out features with non-significant correlations, reducing the list to 275 features. Finally, LASSO regression was applied for further feature selection and dimensionality reduction, narrowing the list to 36 features from T2WI and CE-T1WI, which were then used to construct the stacking ensemble learning model. The results of feature selection can be found in Supplementary Material S1.

### 3.3 Performance assessment of different models

**FIGURE 5:**
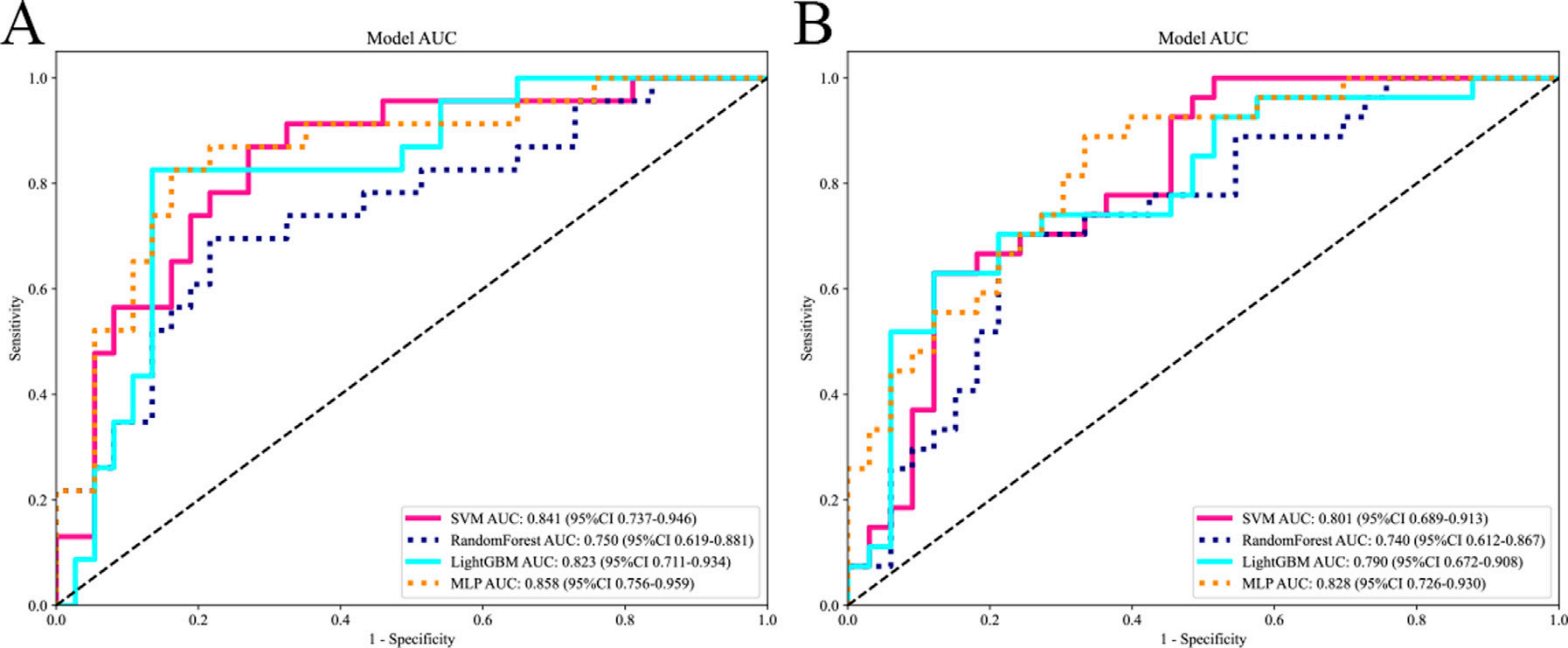
Comparing the AUC of different base models. The subfigures **(A)** and **(B)** respectively display the AUC curves of different base models on the internal and external test sets.

**FIGURE 6:**
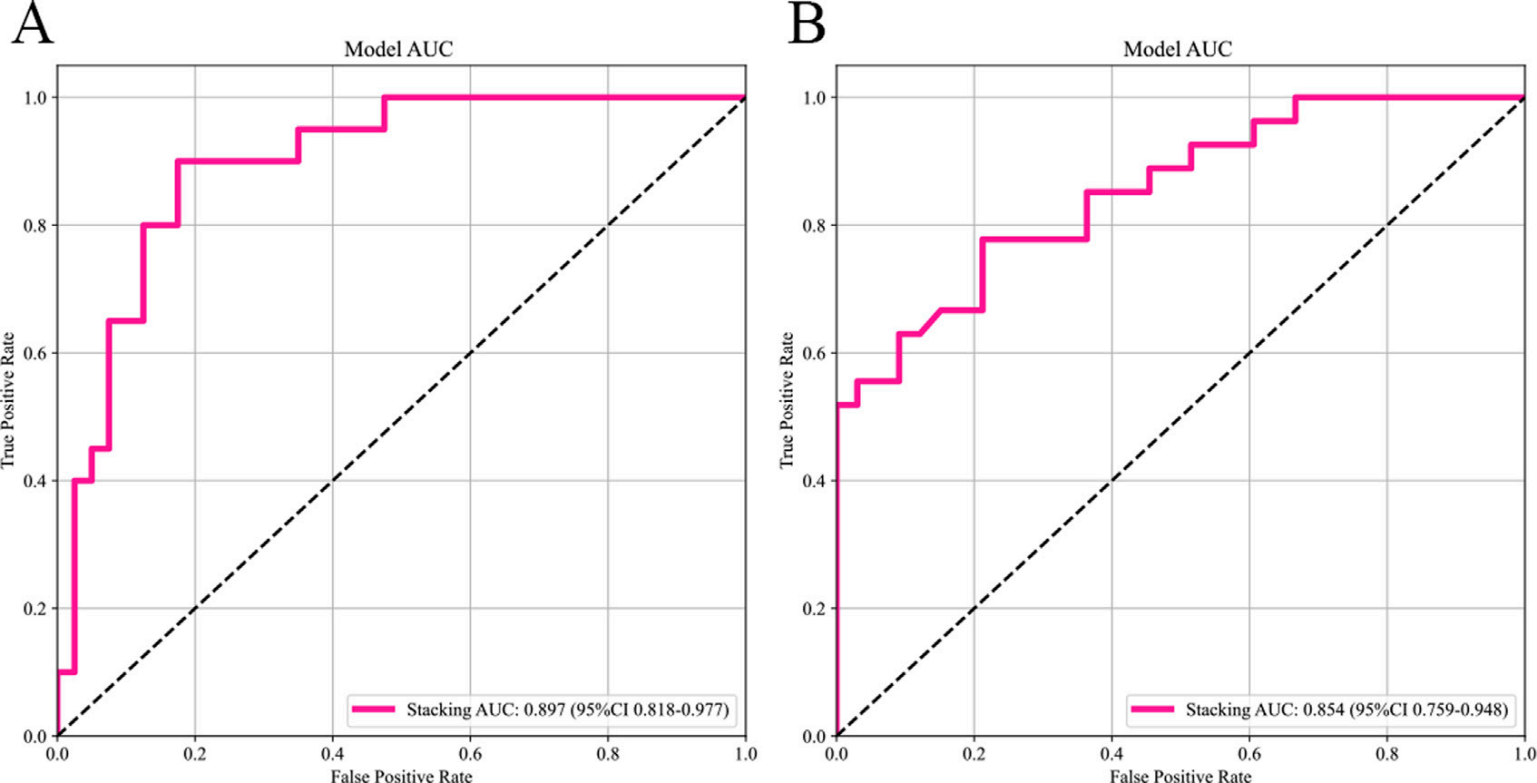
The AUC of the stacking ensemble learning models. Subfigures **(A)** and **(B)** respectively show the AUC curves of the stacking ensemble learning models on the internal and external test sets.

The selected features were utilized to construct models with four base learners. Among these, the MLP model showed superior performance, achieving an AUC of 0.858 (95% CI: 0.756–0.959) on the internal test set and 0.828 (95% CI: 0.726–0.930) on the external validation set. This was followed by SVM, LightGBM, and RF, with internal validation set AUCs of 0.841 (95% CI: 0.737–0.946), 0.823 (95% CI: 0.711–0.934), and 0.750 (95% CI: 0.619–0.881), respectively (Figure 5). Detailed performance evaluations of the models are provided in Table 3. The integration of logistic regression with these four base learners to form an ensemble model led to a substantial enhancement in AUC, reaching 0.897 (95% CI: 0.818–0.977) on the internal test set and 0.854 (95% CI: 0.761–0.952) on the external validation set (Figure 6).

**TABLE 3:** Performance metrics of base models and stacking ensemble models.

		AUC(95%CI)	Accuracy	Sensitivity	Specificity	Precision	P-Value
SVM	Training set	0.949 (0.737–0.946)	0.879	0.856	0.896	0.864	-
	Internal testing set	0.841 (0.737–0.946)	0.767	0.826	0.730	0.655	0.031
	External testing set	0.801 (0.689–0.913)	0.750	0.593	0.879	0.800	0.017
RF	Training set	0.897 (0.859–0.935)	0.824	0.817	0.830	0.787	-
	Internal testing set	0.750 (0.619–0.881)	0.733	0.652	0.784	0.652	<0.01
	External testing set	0.740 (0.612–0.867)	0.717	0.667	0.758	0.692	<0.01
MLP	Training set	0.927 (0.8967–0.958)	0.849	0.837	0.859	0.821	-
	Internal testing set	0.858 (0.756–0.959)	0.817	0.783	0.838	0.750	0.043
	External testing set	0.828 (0.726–0.930)	0.750	0.852	0.667	0.676	0.028
LightGBM	Training set	0.926 (0.893–0.959)	0.858	0.875	0.844	0.812	-
	Internal testing set	0.823 (0.711–0.934)	0.833	0.783	0.865	0.783	0.026
	External testing set	0.727 (0.598–0.8570)	0.683	0.897	0.484	0.619	0.020
Stacking	Internal testing set	0.897 (0.818–0.977)	0.833	0.850	0.825	0.773	-
	External testing set	0.854 (0.761–0.952)	0.767	0.741	0.788	0.741	-

P values were obtained by performing DeLong test between Stacking ensembel model and base models constructed.

## 4 Discussion

In this study, a novel multimodal MRI stacking ensemble learning model was developed and independently validated, utilizing DL based automated segmentation and integrating radiomics data from T2WI and CE-T1WI sequences. A diverse array of machine learning algorithms, including SVM, RF, LightGBM, and MLP, served as base learners, while LR was utilized as the meta-learner to construct the ensemble model. This approach notably enhanced the precision of predicting HIFU ablation efficacy for uterine fibroids. Compared to single-algorithm models, the ensemble model exhibited a marked improvement in performance, with AUC values rising to 0.897 (95% CI: 0.818–0.977) in the internal validation cohort and 0.854 (95% CI: 0.759–0.948) in the external validation cohort, thereby underscoring the model’s superior capability in managing complex radiomics data.

In the field of radiomics, precise image segmentation is crucial as it enhances the accuracy of data analysis and provides a solid foundation for subsequent predictive modeling. Current studies predicting the effectiveness of HIFU therapy often rely on manual contouring (Li et al., 2023; Zheng et al., 2021). This process is not only time-consuming but also prone to human error. Therefore, incorporating automated segmentation technologies is crucial to enhancing efficiency and minimizing human-related biases. In this study, an automated segmentation model based on V-Net was developed specifically for rapid segmentation of uterine fibroids on T2WI and CE-T1WI. This automated tool substantially reduces the need for human intervention, freeing radiomics analysis from the labor-intensive manual contouring and minimizing biases introduced by human factors. This innovation advances the prognostication of HIFU outcomes towards a more efficient and automated future.

Our study has made notable advancements in the field of multimodal MRI analysis by integrating T2WI and CE-T1WI. This integrated approach shows improved predictive performance with an AUC value of 0.858, compared to AUC values of 0.822 for T2WI and 0.848 for CE-T1WI when used independently (Li et al., 2023; Zheng et al., 2021). The multimodal integration strategy effectively leverages the complementary information from different MRI sequences, offering a more comprehensive description of fibroid tissue characteristics and thus enhancing the accuracy of predicting HIFU treatment efficacy. T2WI, with its superior contrast resolution, reveals cellular dense areas and fibrotic regions within fibroids. These regions manifests as high signal intensity on T2WI and may affect ultrasound transmission efficiency, while fibrotic tissue might interfere with ultrasound propagation characteristics, impacting the effectiveness of HIFU treatment (Zhao et al., 2013; Gong et al., 2017). Additionally, T2WI can identify degenerative features such as necrosis, ischemia, edema, and calcification within fibroids, all of which significantly influence treatment outcomes (Andrews et al., 2019). Moreover, CE-T1WI assesses the vascular density and blood flow within fibroids, offering insights into ultrasound energy distribution and the thermal effects of HIFU (Liu et al., 2018). Regions with abundant vasculature may lead to the dissipation of ultrasound energy, thereby reducing treatment efficacy (Lénárd et al., 2008). Nevertheless, traditional MRI image analysis faces challenges related to subjective interpretation and difficulties in detecting subtle signal intensity variations. This study addresses these limitations by introducing radiomics methods, which overcome these issues by extracting a large number of quantitative features from MRI scans, thereby offering an objective and precise description of fibroid structural heterogeneity (Gillies et al., 2016; Lambin et al., 2017). These features not only enhance the analysis of fibroid morphology but also improve predictive capabilities for HIFU treatment outcomes. Furthermore, the combined use of multimodal MRI radiomic features demonstrates the complementarity among different MRI modalities, which is crucial for revealing the biological characteristics of fibroids.

In the medical field, where precise treatment is essential, the efficient and comprehensive utilization of multimodal MRI data is critical for enhancing predictive model performance. However, conventional single machine learning algorithms often fail to fully harness the potential of these invaluable multimodal radiomics datasets due to their disparate methodologies in feature handling and focal points. To address this challenge, this study employs four machine learning algorithms, each with a distinctive modeling philosophy: SVM, RF, MLP, and LightGBM, as base learners. SVM excels in managing small sample sizes and nonlinear challenges by identifying optimal hyperplanes within complex feature spaces (Cortes and Vapnik, 1995); RF enhances model stability and mitigates overfitting through ensemble decision trees and a voting mechanism (Breiman, 2001); LightGBM demonstrates exceptional performance in large-scale data processing by iteratively refining weak classifiers (Ke et al., 2017); and MLP leverages deep neural networks to capture intricate nonlinear features, thereby augmenting feature learning capacity (Rumelhart et al., 1986). The study integrated these base models using a meta-learner, LR, to construct a stacked ensemble learning framework. This sophisticated approach not only amalgamates the strengths of diverse models but also rectifies the limitations inherent in individual algorithms with regard to multimodal MRI radiomics features utilization. Consequently, it more effectively utilizes multimodal MRI radiomic features to improve both the accuracy and stability of preoperative predictions for HIFU treatment. This robust and advanced tool improves the precision of preoperative assessments, supports personalized treatment strategies, and is expected to enhance treatment decision-making and patient management in clinical practice.

### 4.1 Limitations

There are some limitations in this study. First, the sample size is relatively small. Future studies should include a larger cohort of patients to enhance the validity of the findings. Second, the effect of HIFU for uterine fibroids is affected by multiple factors, and future studies should integrate more clinical indicators to further improve the performance and credibility of the model.

## 5 Conclusion

This study developed a radiomics stacking ensemble model based on multimodal MRI, incorporating automatic segmentation techniques to predict the efficacy of HIFU ablation for uterine fibroids. It offers a comprehensive method for quantifying uterine fibroid heterogeneity and serves as a more precise supplementary tool for clinical practice.

## Data Availability

The raw data supporting the conclusions of this article will be made available by the authors, without undue reservation.
