# Supplementary figures and images for: Multimodal MRI radiomics-based stacking ensemble learning model with automatic segmentation for prognostic prediction of HIFU ablation of uterine fibroids: a multicenter study
Source: Front Physiol. 2024 Dec 20;15:1507986. doi: 10.3389/fphys.2024.1507986 (PMC11695313; doi:10.3389/fphys.2024.1507986)

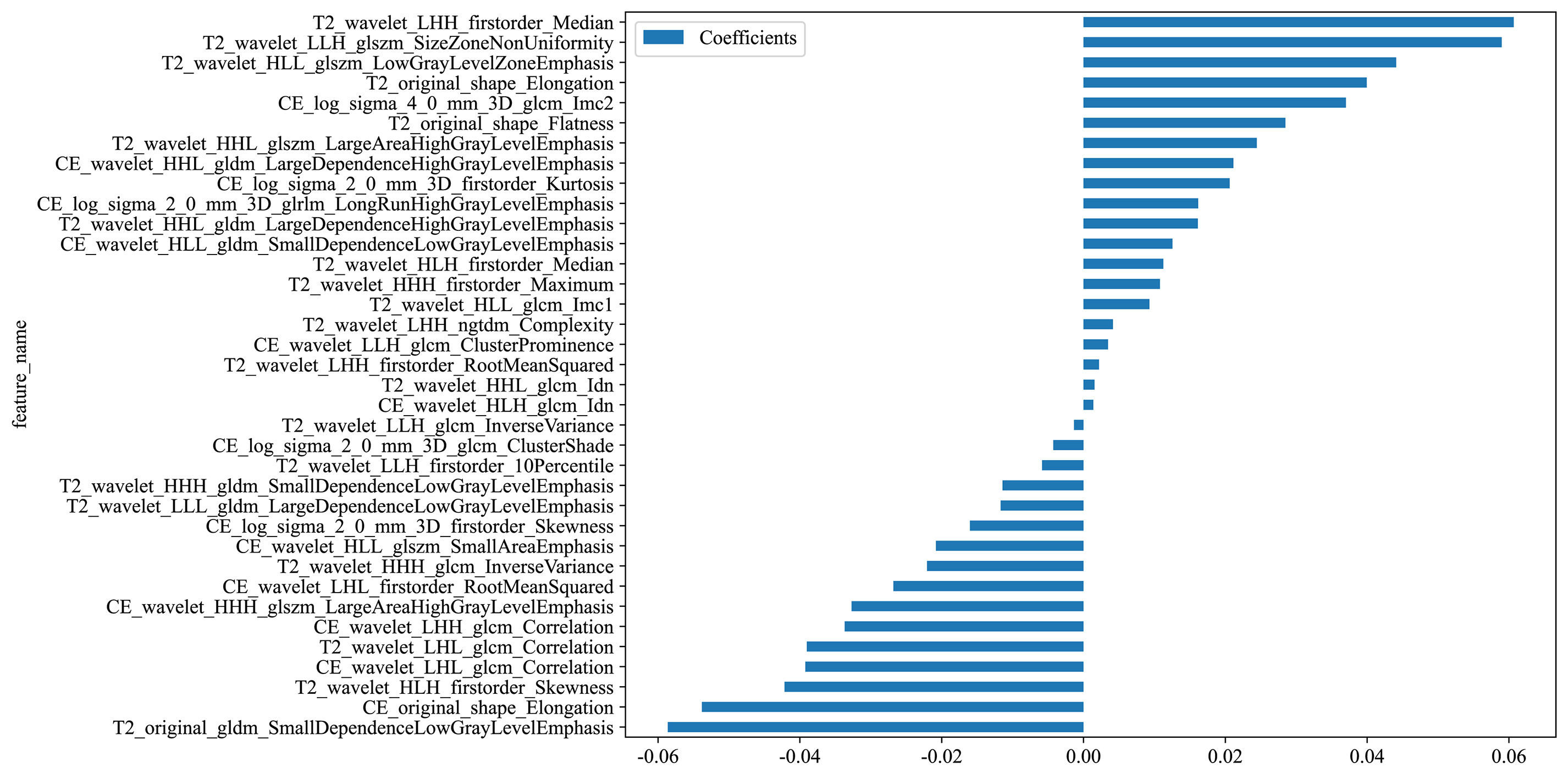

Supplement: Supplementary file 1 [file Image1.tif]
